# Exploring the potential of comparative *de novo* transcriptomics to classify *Saccharomyces* brewing yeasts

**DOI:** 10.1371/journal.pone.0238924

**Published:** 2020-09-23

**Authors:** Jürgen Behr, Meike Kliche, Andreas Geißler, Rudi F. Vogel

**Affiliations:** Lehrstuhl für Technische Mikrobiologie, Technische Universität München, Freising, Germany; CNR, ITALY

## Abstract

In this work the potential of comparative transcriptomics was explored of *Saccharomyces* (*S*.) *cerevisiae* and *S*. *pastorianus* for their discrimination. This way an alternative should be demonstrated to comparative genomics, which can be difficult as a result of their aneuoploid genomes composed of mosaics of the parental genomes. Strains were selected according to their application in beer brewing, i.e. top and bottom fermenting yeasts. Comparative transcriptomics was performed for four strains each of commercially available *S*. *cerevisiae* (top fermenting) and *Saccharomyces pastorianus* (bottom fermenting) brewing yeasts grown at two different temperatures to mid-exponential growth phase. A non-reference based approach was chosen in the form of alignment against a *de novo* assembled brewery-associated pan transcriptome to exclude bias introduced by manual selection of reference genomes. The result is an analysis workflow for self-contained comparative transcriptomics of *Saccharomyces* yeasts including, but not limited to, the analysis of core and accessory gene expression, functional analysis and metabolic classification. The functionality of this workflow is demonstrated along the principal differentiation of accessory transcriptomes of *S*. *cerevisiae versus S*. *pastorianus* strains. Hence, this work provides a concept enabling studies under different brewing conditions.

## Introduction

### Brewing and yeasts

The brewing of beer is a chemically complex and lastly highly controlled biotechnological process. According to the German brewers’ association there are more than 40 malt varieties, 250 different hops and over 400 yeast strains to choose from, not to mention the staggering differences in water quality. On the contrary, most of the beers result from fermentation with a very limited number of yeast strains, e.g. in Germany mostly four strains with one single strain accounting for approximately 65% are used. Thus, the unexploited combinations and possibilities to use different yeasts strains for the development of new beers are virtually endless [1].

*Saccharomyces* brewing yeasts can be categorised into the two species *Saccharomyces* (*S*.) *cerevisiae* and *S*. *pastorianus*. *S*. *cerevisiae* strains are fermented at elevated temperatures to produce top fermented beer styles, such as ales, stouts, wheat beer, German Alt and Kölsch. In contrast *S*. *pastorianus* yeasts are used to produce bottom fermented beer styles, such as lager, Pilsner and Export, at lower temperatures over a longer fermentation time.

Ale yeast is the oldest microbe actively employed by mankind to refine raw materials into food and drinks with archaeological evidence of beer brewing and wine making dating back to the pre-pottery neolithical A (9.500 – 8.800 BC) [2]. After centuries of being held in artificial environments and being domesticated for particular applications, today there are highly specific *S*. *cerevisiae* strains deployed for the production of the different top fermented beer styles. Typical yeasts of this group are the phenolic off-flavour positive (POF^(+)^) TUM68, Germany´s most prevalent wheat beer yeast; the POF^(-)^ TUM177, the most important Kölsch yeast and TUM211 (POF^(-)^) and TUM511 (POF^(+)^) as two important English and American ale yeasts, respectively.

*S*. *pastorianus* lager yeasts belong to two distinct lineages that originated from two separate hybridisation events of *S*. *cerevisiae* with the only relatively recently identified *Saccharomyces eubayanus* [3]: group I Saaz-type yeast and group II Frohberg-type yeasts [4, 5]. They were first phenotypically and from the practician´s point of view described as exhibiting clearly different fermentation behaviour by Noonan in 1996 [6]. The Saaz group strains, named after the area they were mainly used in, which is now in the Czech Republic, are triploid(-like), are better adapted to cold growth conditions [7] and show a lower concentration of aroma compounds, such as ethyl acetate, isoamyl alcohol and isoamyl acetate [8]. The most famous member of the Saaz group strains is the *S*. *carlsbergensis* type strain CBS 1513, which is included in this study along with the *S*. *monacencis* type strain CBS 1503. The Frohberg group strains were generally used in Dutch and Danish breweries other than the Carlsberg brewery, they are tetraploid(-like), ferment faster than the Saaz group strains and show a greater aroma richness [8, 9]. TUM34/70, one of the most used lager strains in Europa [10], is the major representative of the Frohberg group and along with TUM66/70 included in this study. It should be mentioned that these strains are triploid(-like) and tetraploid(-like) only in terms of DNA content (see e.g. [11]). In fact they all are aneuploids having chimeric genomes composed of mosaics of the parental genomes. For over ten years the genetic diversity of brewing yeasts has been cause for discussion. The declarations range from higher genetic diversity in ale yeasts compared to the more conserved genomes of lager yeasts [12], over a low genetic variation between *S*. *cerevisiae* isolates [11] to only two separate interspecies hybridization events in the formation of *S*. *pastorianus* yeasts as cause for their relatively limited aroma diversity compared to *S*. *cerevisiae* yeasts, even though *S*. *pastorianus* yeasts form two distinct phenotypic groups and *S*. *cerevisiae* yeasts do not [8, 13]. This illustrates why the identification and classification of yeasts on the genomic level can result in misclassification and a non-reliable prediction of fermentation behaviour. Applied comparative transcriptomics may enable closure of this knowledge gap [14].

### Transcriptomics of brewing yeasts

While the analysis of genetic variation addresses the question of the hypothetical abilities of an organism, transcriptomics pursues only the definite expression independently of that part or the genomic potential, which remains unexpressed under the conditions given. This is especially relevant in settings where the interaction of the microorganism with its environment dictates the desired outcome, e.g. in beer brewing.

Many studies employing microarray technology have been conducted to gain insights into the fermentative behaviour of brewing yeasts, to elucidate their aroma forming capabilities and to characterize defining beer type properties. Microarrays are a well-established technology and powerful and cost-effective tools for definite questions. Next generation sequencing (NGS) techniques, such as RNA-Seq, could even be applied independently of a reference genome for the study of species without a fully sequenced genome, since it directly accesses the base sequence [15]. To the best of our knowledge no investigative effort has been made to compare the gene expression profiles of top and bottom fermenting brewing yeasts, neither employing microarray technology, nor RNA sequencing.

### Aim of the study

In this study the potential of comparative transcriptomics was explored of *S*. *cerevisiae* and *S*. *pastorianus* for their discrimination, and to avoid difficulties in the comparison of their aneuoploid genomes composed of mosaics of the parental genomes. The use of these yeasts in typical brewing processes employs different temperatures (15 or 20°C). Still, for comparative transcriptomics targeted at differences useful for yeast classification, the transcriptional response to sub-optimal temperature for the respective yeast strain must be determined and possibly excluded. Therefore, comparative transcriptomics was performed in parallel at 15°C and 20°C for all cultures of four yeast strains each belonging to the species *S*. *cerevisiae* and *S*. *pastorianus*. Statistical analyses, alignment of pre-processed reads to a *de novo* assembled brewery associated pan transcriptome and functional annotation were performed to identify the core, pan and accessory transcriptome.

In order to accomplish that we aimed to establish a flexible workflow to perform self-contained transcriptomics and as a proof-of-concept evaluate the following hypothesis:

If ale yeasts are genetically more diverse than lager yeasts [12] and lager yeasts can be phenotypically placed into two distinctly different groups [4, 5], then this inherent diversity should be visible through transcriptomic analysis.

## Materials and methods

### Yeast strains and culture media

The *S*. *cerevisiae* strains used in this study were: TUM 68, the most widely used commercial wheat beer strain in Germany; TUM 177, used for brewing Kölsch beer; TUM 211, an English ale strain, and TUM 511, an American ale strain, which is phylogenetically related to wine yeast strains [16]. The *S*. *pastorianus* strains used in this study were: TUM 34/70, one of the most widely used lager strains in Europe and representative of the group II Frohberg type *S*. *pastorianus* yeasts, as well as TUM 66/70, another Frohberg yeast; CBS 1503, the *S*. *monacencis* type strain and a group I Saaz type yeast, as well as CBS 1513, the *S*. *carlsbergensis* type strain and another Saaz type yeast. The strains were partly available from the in-house strain collection and partly kindly provided by the Forschungszentrum Weihenstephan für Brau- und Lebensmittelqualität (BLQ, Freising, Germany). The yeast strains were prepared as glycerol stocks (12% (*w/v*)) at the beginning of the project and stored at -80°C as strains (isogenic strains in brackets) TMW 3.250 (TUM 86), TMW 3.256 (TUM 177), TMW 3.261 (TUM 211), TMW 3.673 (TUM 511), TMW 3.275 (TUM 34/70), TMW 3.285 (TUM 66/70), TMW 3.287 (CBS 1503) and TMW 3.681 (CBS 1513). Cultures were grown at 30°C on YPD agar containing 2% glucose (*w/v*), 1% peptone (*w/v*), 5% yeast extract (*w/v*) and 1,5% agar (*w/v*) and propagated in liquid YPD (2% glucose (*w/v*), 1% peptone (*w/v*), 5% yeast extract (*w/v*)). For propagation 50 mL YPD were inoculated with a single colony from the second of two subsequent YPD agar plates and grown aerobically for three days at 30°C and 180 rpm.

### Growth and sample preparation

All experiments were conducted in biological triplicates. The growth experiments were performed in 250 mL Erlenmeyer flasks containing 50 mL YPD and yeast cultures pitched at cell densities of 3 × 10^5^ cells mL^-1^. Cells were grown at 15°C and 20°C, up to mid-exponential growth phase, which was determined in a previous experiment for all strains and both temperatures. These temperatures were chosen as the most frequently applied process temperatures for bottom and top fermented beers, respectively. Cells were then counted using a Thoma chamber (depth = 0.1 mm, volume = 0.0025 mm^2^) and an aliquot of 3 × 10^8^ cells was treated with RNAlater (Invitrogen, NN Bleiswijk, Netherlands), flash frozen in liquid nitrogen and stored at -80°C until RNA isolation. Total RNA was extracted from the frozen cells through mechanical disruption with acid washed glass beads and a bead mill (FastPrep-24, MP Biomedicals, Irvine, CA, USA) and high quality RNA was isolated using the RNeasy Kit from Qiagen (Qiagen, Hilden, Germany). The quality and the quantity of the isolated RNA were checked by using Nanodrop 1000 spectrometer (Peqlab Biotechnologie GmbH, Erlangen, Germany) and isolated RNA was stored at -20°C until shipment. During shipment the samples were cooled on dry ice.

### Sequencing

RNA was submitted to a commercial provider for library construction and sequencing. Illumina HiSeq sequencing with a read length of 2 × 150 bp (paired-end reads) was carried out by GATC Biotech (Konstanz, Germany).

### *In silico* analyses

A tailored bioinformatics workflow was developed to process and analyse the paired-end Illumina sequences. For that several established tools and algorithms were employed. The general workflow, which is detailed below, included:

1. Processing of raw read data of every single strain
2. Compilation of a *de novo* meta transcriptome using SPAdes [17]
3. Normalisation of read data
4. Alignment, assembly and calculation of differential expression using the Tuxedo package [18]:
  - Alignment of pre-processed reads to the transcriptome using tophat2 with Bowtie2 as alignment engine
  - Assembly of accepted hits for each replicate into transcripts using cufflinks and cuffmerge
  - Calculation of differential expression using cuffdiff
5. Functional annotation
  - Extraction of sequences (of differentially expressed genes)
  - BLAST of gene sequences against MIPS Functional Catalogue (provided as supplementary material—S1 File).
6. Statistical analysis

### Processing of transcriptomic raw data

Trimming and lengthsorting of reads was performed using the paired-end reads-aware trimming algorithm SolexaQA [19] and resulted in high quality reads in FASTQ format.

The resulting preprocessed data sets are deposited in the European Nucleotide Archive of the EMBL-EBI. The accession number of the whole project is PRJEB33088. The sample accession numbers are ERS3526866-913.

### Compilation of a *de novo* meta transcriptome

A de novo pan transcriptome was compiled using rnaSPAdes with all parameters set to their default values [17].

### Normalisation of read data

The calculation of the normalisation factors was performed using the R package DESeq with R software (DESeq version 1.30.0, R software version 3.4.3 “Kite-Eating Tree”, http://www.r-project.org) [20]. Samtools (version 0.1.19_44428cd., http://www.htslib.org) and the function bamCompare from the software package deepTools (version 2.5.3, https://deeptools.readthedocs.io) were used to apply the normalisation factors to the gene counts stored in the alignment files. Differential gene expression was calculated based on the normalised gene counts (see above).

### Alignment, assembly and calculation of differential expression using the Tuxedo package

Alignment to the *de novo* assembled pan transcriptome, transcriptome assembly and calculation of the differential gene expression was done using the Tuxedo protocol [18].

For alignment and subsequent transcriptome analysis a non-genomic-reference based approach was chosen, this means that each set of raw reads was aligned to the *de novo* assembled pan transcriptome and transcripts were assembled accordingly.

The trimmed and pre-processed paired-end reads were aligned to the pan transcriptome using TopHat2 version 2.1.1 [21] with Bowtie2 version 2.3.2 as its read-alignment engine [22] with all parameters set to their default values. Mapping statistics can be found in Table 1: Read alignment statistics. The read alignments were normalised (see below) and further assembled into transcripts using Cufflinks v2.2.1 [23] and merged into a merged transcriptome using Cuffmerge v1.0.0. Cuffdiff v2.2.1 was used to calculate the differential gene expression and graphic output was generated using the R packages CummeRbund version 2.18.0 [24] with R software version 3.4.0 (“You Stupid Darkness”, http://www-r-project.org), ggplot2 version 2.2.1 [25] and factoextra version 1.0.5.

**Table 1:** Read alignment statistics.

			15°C		20°C	
	sample	replicate	CPAR*	ORMR**	CPAR*	ORMR**
*Saccharomyces cerevisiae*	TMW 3.250	1	67.5	80.8	68.3	82.2
		2	69.1	82.6	67.4	81.5
		3	66.4	79.5	67.2	81.9
	TMW 3.256	1	70.5	83.7	63.4	76.7
		2	65.2	79.6	63.3	75.5
		3	62.9	77.0	62.2	76.6
	TMW 3.261	1	59.3	71.8	62.8	75.7
		2	64.0	77.9	64.6	78.5
		3	61.9	75.4	61.1	73.8
	TMW 3.673	1	64.2	76.9	55.6	67.6
		2	62.7	74.1	65.0	78.2
		3	64.1	76.4	64.2	77.5
*Saccharomyces pastorianus*	TMW 3.275	1	68.9	82.3	72.1	84.6
		2	66.1	78.2	62.7	74.6
		3	68.0	80.9	61.4	72.7
	TMW 3.285	1	66.3	78.2	71.9	83.6
		2	67.5	79.8	63.8	75.9
		3	65.9	78.7	69.7	82.5
	TMW 3.287	1	68.3	81.3	66.9	79.0
		2	67.9	80.5	70.9	83.1
		3	70.2	83.0	71.3	83.8
	TMW 3.681	1	68.1	82.6	68.9	83.0
		2	68.0	83.7	68.5	82.7
		3	68.4	84.0	68.1	81.8

*CPAR: concordant pair alignment rate (%), obtained with TopHat v2.1.1, using bowtie 2.3.2 with default settings.

**ORMR: overall read mapping rate (%), obtained with TopHat v2.1.1, using bowtie 2.3.2 with default settings.

### Functional annotation

Sequences of differentially expressed genes were extracted from the Cuffdiff output, translated to protein sequences and blasted against the MIPS Functional Catalogue [26, 27] (provided as supplemental data—S1 File).

### Statistical analysis

All experiments were performed in triplicates. Statistical analyses were performed using R software version 3.4.3 “Kite-Eating Tree”. Principal component analysis was performed using the DESeq package for R (version 1.30.0) after compiling the data in Perseus version 1.6.0.7.

Principal component analysis with a false discovery rate (FDR) of 0.05 was conducted in Perseus version 1.6.0.7.

### Functional linking to metabolic traits

As the aim of this study was to principally differentiate top from bottom fermenting yeasts as a proof of concept for the functionality of the workflow, and regulatory effects resulting from different growth temperatures should be excluded. In this context, a gene was considered as expressed in a strain if the mean values of all three replicates at both temperatures were greater than zero. This approach excludes the temperature bias and therefore regulatory effects from the evaluation. Gene expression is given in FPKM values calculated by the Tophat2 algorithm. The overlapping and strain specific gene expression of all four *S*. *cerevisiae* and *S*. *pastorianus* strains, respectively, was determined through depiction with Venn diagrams using Venny [28]. The genes mutually expressed in all four strains of each species were again compared via Venn diagram to assess the core transcriptome of all eight investigated brewing yeast strains. For metabolic inspection the genes were then sorted into functional categories according to the MIPS Functional Catalogue [26, 27].

## Results and discussion

This work was conducted to demonstrate the potential of comparative transcriptomics to delineate brewing yeast types despite their complex chromosomal settings. While the data should have limited significance with respect to their behaviour or metabolism in the fermentative state of the brewing process, they instead should demonstrate the differentiative potential of the self-contained *de novo* transcriptomics approach to differentiate these yeasts independently from brewing experiences.

**Fig 1:**
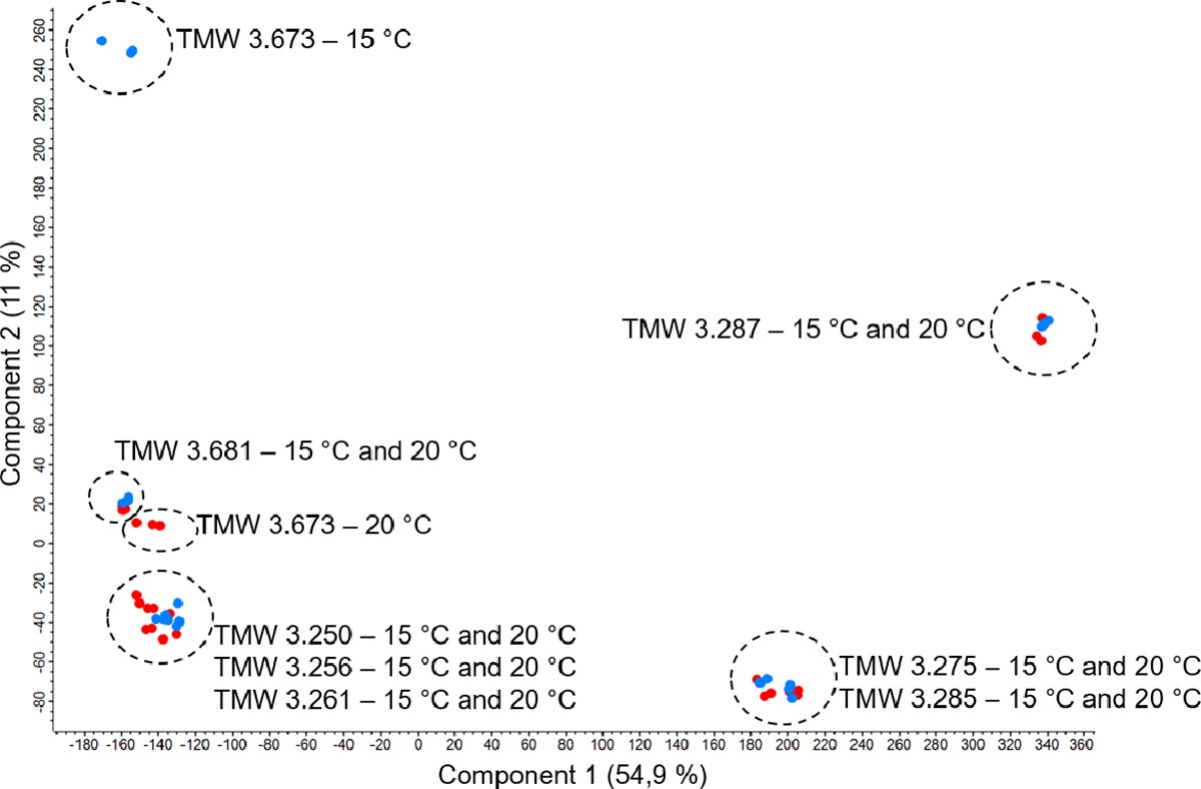
PCA of read counts of eight brewing yeasts, Benjamini-Hochberg corrected, FDR = 0.05. Blue dots represent 15°C, red dots represent 20°C data. *S*. *cerevisiae* strains TMW 3.250 (TUM 86), TMW 3.256 (TUM 177), TMW 3.261 (TUM 211), TMW 3.673 (TUM 511); *S*. *pastorianus* strains (Frohberg) TMW 3.275 (TUM 34/70), TMW 3.285 (TUM 66/70), (Saaz) TMW 3.287 (CBS 1503) TMW 3.681 (CBS 1513).

The results of the principal component analysis of all transcripts are shown in Fig 1. The *S*. *pastorianus* yeasts form three distinct clusters, whereby the two Frohberg yeasts form one cluster and the two Saaz yeasts diverge from that cluster and from one another into opposing directions. The cultivation temperatures used in this study had no effect on the expression profile of all four strains. This is to be seen differently from an exposure to cold shock upon yeast storage, which resulted in distinct responses in e.g. *S*. *pastorianus* [29]. The *S*. *cerevisiae* strains form a single cluster with the exception of the American ale yeast TUM511, which also is the only one showing significant temperature dependent differences in the gene expression profile.

*S*. *pastorianus* yeasts are reported to possess limited genetic diversity and therefore a limited influence on the final flavour profile of bottom fermented beers [30], especially in contrast to the enormous genetically encoded aroma producing ability of top fermenting *S*. *cerevisiae* yeasts [31–33]. This is not replicated in the clustering based on the expression profiles and may imply that parts of highly homologous genome regions are not (differentially) expressed under the conditions chosen for our experiment.

**Fig 2:**
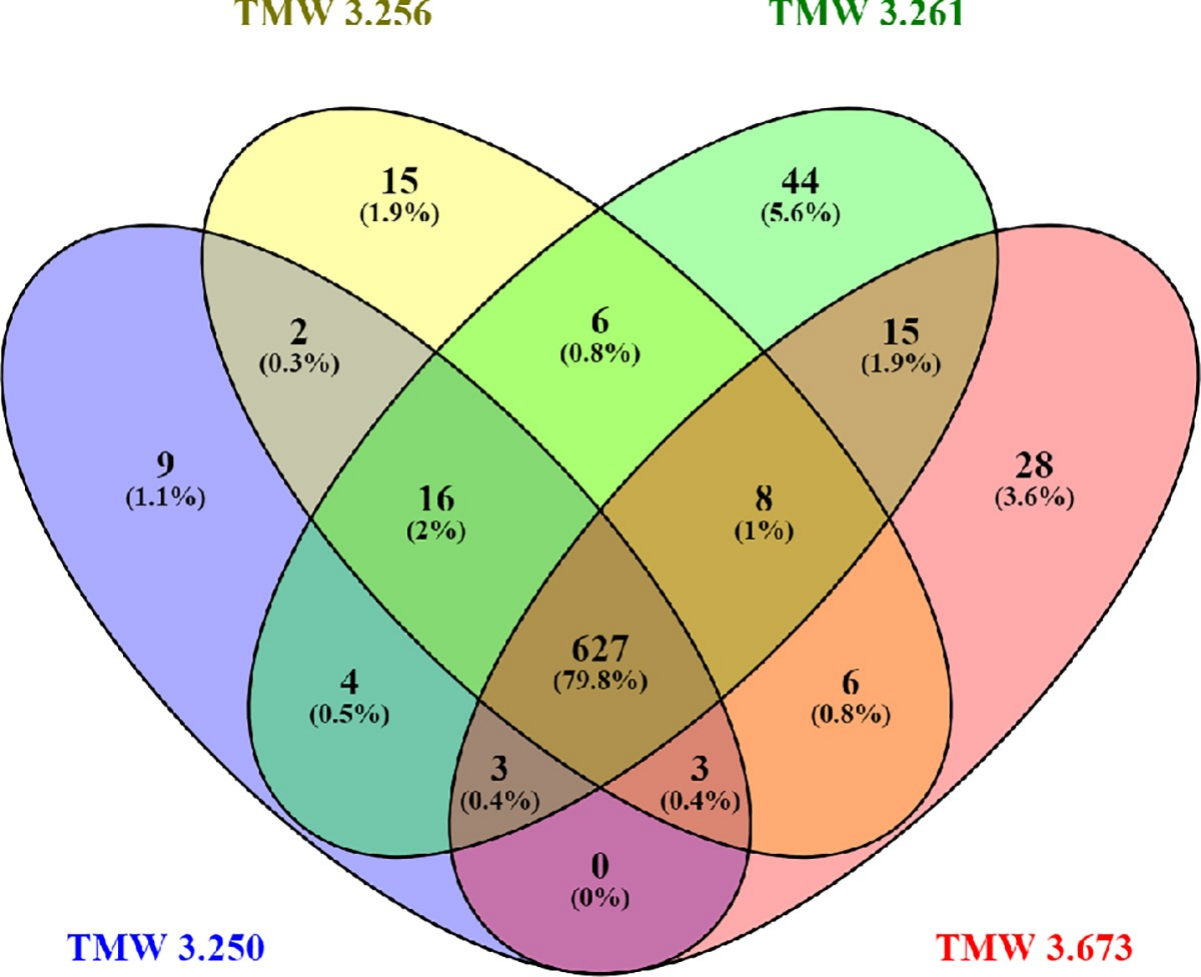
Venn diagram of the expressed genes of four *S*. *cerevisiae* strains. The Venn diagram was generated using Venny [28].

**Fig 3:**
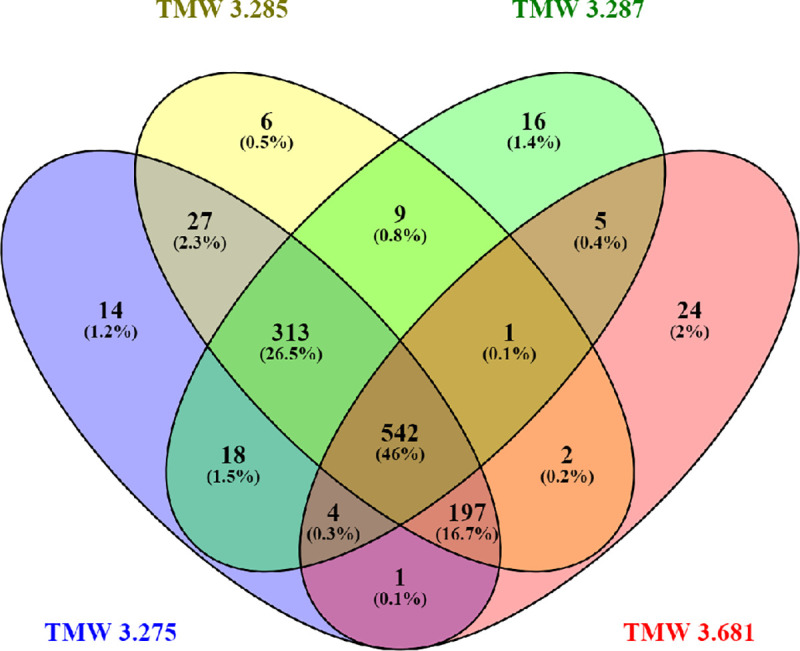
Venn diagram of the expressed genes of four *S*. *pastorianus* strains. The Venn diagram was generated using Venny [28].

All expressed transcripts of one species, that could be annotated with a gene name according to the MIPS Functional Catalogue, were assessed using Venn diagrams [28]. This meant 786 transcripts expressed in *S*. *cerevisiae* yeasts and 1179 transcripts expressed in *S*. *pastorianus* yeasts. As shown in Fig 2 of the 786 transcripts expressed in *S*. *cerevisiae*, 627 genes are common to all four strains. This corresponds to 79.8% of the transcripts. In *S*. *pastorianus* only 542 of the 1179 transcripts are common to all four strains, which corresponds to 46% (Fig 3). As indicated by the respective numbers in these figures the strain specific in-group diversities were low. Generally, the (unexpectedly) higher number of transcripts found in the *S*. *pastorianus* group may be attributed to the presence of orthologues from two species (*S*. *cerevisiae* and *S*. *eubayanus*) in its genome. This shows that the inherit diversity in ale and lager yeasts is detectable through transcriptomic analysis, in which Ale yeasts presented themselves as less diverse compared to lager yeasts.

For *S*. *cerevisiae* none of the other transcript groups in the Venn diagram exceeded 6%, with the transcripts unique in the English ale yeast strain TMW 3.361 forming the biggest group with 44 transcripts (5.6%). The wheat beer yeast strain TMW 3.250 has only nine (1.1%) unique transcripts, the Kölsch yeast strain TMW 3.265 has only 15 (1.9%) unique genes and the American ale yeast strain TMW 3.673 28 (3.6%). This is shown in Fig 2. Fig 3 shows that two bigger groups are found within the *S*. *pastorianus* yeasts: the three strains TMW 3.275, TMW 3.285 and TMW 3.287 have 313 genes in common, this corresponds to 26.5%, whereas the three strains TMW 3.275, TMW 3.285 and TMW 3.681 share 197 genes, which corresponds to 16.7%. However, the number of transcribed genes for *S*. *pastorianus*, which are unique to one strain, is small: 14 (1.2%) and 6 (0.5%) for the two Frohberg yeasts TMW 3.275 and TMW 3.285, respectively, 16 (1.4%) for the Saaz yeast TMW 3.287 and 24 (2%) for the *S*. *carlsbergensis* type strain TMW 3.681.

**Fig 4:**
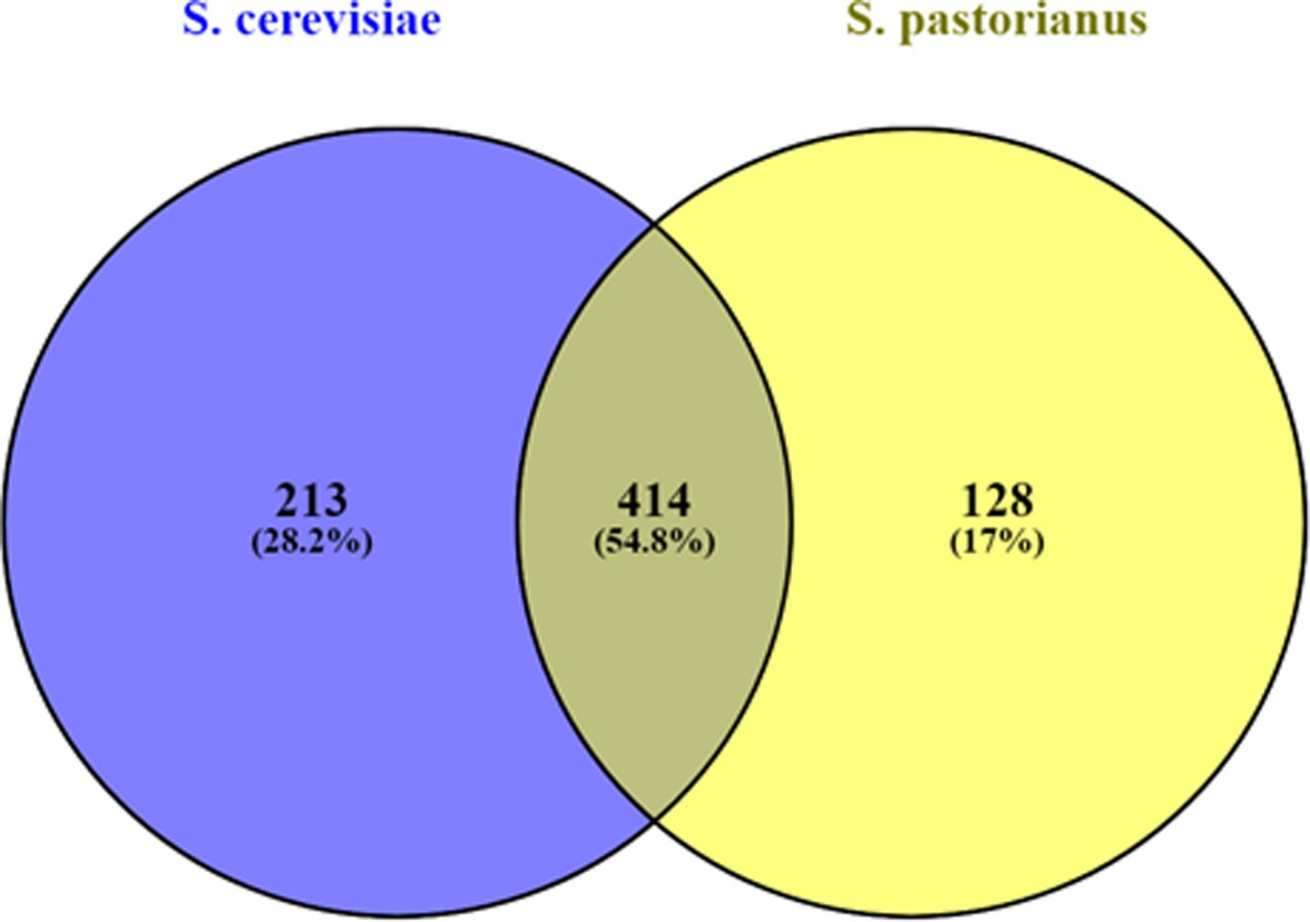
Venn diagram showing the overlap of 627 genes expressed by all four *S*. *cerevisiae* (Fig 2) and 542 genes expressed by all four *S*. *pastorianus* (Fig 3) strains, respectively. The Venn diagram was generated using Venny [28].

In Fig 4 the comparison of the 627 transcripts common in all four *S*. *cerevisiae* strains and of the 542 transcripts common in all four *S*. *pastorianus* strains is shown. All eight *Saccharomyces* brewery related strains share 414 transcribed genes, which corresponds to 54.8%. The remaining transcripts are split into 213 (28.2%) for *S*. *cerevisiae* and 128 (17%) for *S*. *pastorianus*. These transcripts were classified according to their MIPS Functional Categories, and the ones common to all eight strains are summarised in Table 2. The genes exclusively expressed in either all four *S*. *cerevisiae* or all four *S*. *pastorianus* strains are shown in Tables 3, 4 and 5. Categories high in transcript numbers for all eight strains as well as genes exclusively expressed in either *S*. *cerevisiae* or *S*. *pastorianus* strains belong to categories reflecting transcription and translation, such as protein binding, ribosomal proteins, transcriptional control or translation, or categories related to metabolic activities, such as phosphate metabolism, C-compound and carbohydrate metabolism, and lipid, fatty acid and isoprenoid metabolism. This shows that *S*. *cerevisiae* and *S*. *pastorianus* yeasts, when analysed under the same experimental conditions, display different solutions on the gene expression level to fulfill similar metabolic requirements.

**Table 2:** Categorisation of genes expressed in all eight examined strains. Shown are the 20 MIPS FunCat categories with the highest gene count.

MIPS functional category	Gene count	Gene names
protein binding	114	ANB1, APC4, APM2, ARF2, ASF1, BMH1, BMH2, BNR1, CCT6, CDC27, CDC33, CHC1, CIC1, CMP2, COX2, CPR5, DED1, EFT1, EMP24, ERB1, FIN1, HSP31, HTA2, HTB1, LSM2, MCM6, NMD2, NRG1, PAN1, PCF11, PEP1, PFY1, PPH21, PPT1, PRE3, PRP8, RNR4, RPA135, RPB5, RPL10, RPL11A, RPL13A, RPL15A, RPL16A, RPL16B, RPL18A, RPL18B, RPL19A, RPL19B, RPL1B, RPL20B, RPL21A, RPL21B, RPL22A, RPL22B, RPL24A, RPL25, RPL2A, RPL3, RPL30, RPL31A, RPL32, RPL35A, RPL35B, RPL36B, RPL37A, RPL38, RPL39, RPL42B, RPL43B, RPL4A, RPL8B, RPL9B, RPN10, RPP0, RPS10A, RPS11B, RPS14B, RPS16A, RPS16B, RPS17A, RPS1B, RPS2, RPS20, RPS22A, RPS26A, RPS27B, RPS28A, RPS3, RPS31, RPS4A, RPS4B, RPS5, RPS8B, RPS9B, SAR1, SIR4, SLY1, SNU114, SSZ1, SUP45, TIF1, TMA19, TSR1, TUB2, UBC4, UBR1, UFD2, UFD4, VMA10, VMA3, YPT52, YRB1, ZUO1
ribosomal proteins	63	MAK21, MRP13, MRPL35, MRPL44, RPL10, RPL11A, RPL13A, RPL15A, RPL16A, RPL16B, RPL18A, RPL18B, RPL19A, RPL19B, RPL1B, RPL20B, RPL21A, RPL21B, RPL22A, RPL22B, RPL24A, RPL25, RPL29, RPL2A, RPL3, RPL30, RPL31A, RPL32, RPL35A, RPL35B, RPL36B, RPL37A, RPL38, RPL39, RPL42B, RPL43B, RPL4A, RPL6B, RPL8B, RPL9B, RPP0, RPP1B, RPP2B, RPS10A, RPS11B, RPS14B, RPS16A, RPS16B, RPS17A, RPS1B, RPS2, RPS20, RPS22A, RPS26A, RPS27B, RPS28A, RPS3, RPS31, RPS4A, RPS4B, RPS5, RPS8B, RPS9B
translation	62	ALA1, ANB1, CPR5, EFT1, GCD11, MSS1, RPL10, RPL11A, RPL13A, RPL15A, RPL16A, RPL16B, RPL18A, RPL18B, RPL19A, RPL19B, RPL1B, RPL21A, RPL21B, RPL22A, RPL22B, RPL24A, RPL25, RPL2A, RPL3, RPL30, RPL31A, RPL32, RPL35A, RPL35B, RPL36B, RPL37A, RPL38, RPL39, RPL43B, RPL4A, RPL8B, RPL9B, RPP0, RPS10A, RPS11B, RPS14B, RPS16A, RPS16B, RPS17A, RPS1B, RPS2, RPS20, RPS22A, RPS26A, RPS27B, RPS28A, RPS3, RPS31, RPS4A, RPS4B, RPS5, RPS8B, RPS9B, SNU114, SUP45, TIF1
ribosome biogenesis	53	CRM1, ERB1, MAK21, RNA1, RPL11A, RPL13A, RPL15A, RPL16A, RPL16B, RPL18A, RPL18B, RPL19A, RPL19B, RPL1B, RPL22A, RPL22B, RPL24A, RPL25, RPL2A, RPL31A, RPL32, RPL35A, RPL35B, RPL36B, RPL37A, RPL38, RPL39, RPL42B, RPL43B, RPL4A, RPL8B, RPL9B, RPP1B, RPP2B, RPS11B, RPS14B, RPS16A, RPS16B, RPS1B, RPS2, RPS20, RPS22A, RPS26A, RPS27B, RPS28A, RPS3, RPS31, RPS4A, RPS4B, RPS5, RPS8B, RPS9B, TSR1
transcriptional control	37	ARP9, ASF1, ASG1, BDF2, BMH1, BMH2, CAF40, CCR4, CHD1, CMP2, DED1, FKH1, FZF1, HHF1, HTA2, HTB1, IBA57, JHD2, MAL33, MCM6, NOT5, ORC3, REG2, RIF1, RSC30, SIN3, SIR4, SPT5, SRB2, STB2, SUS1, SWI3, THI20, UBC4, UBP10, VHS3, WTM2
phosphate metabolism	36	ARF2, CDC15, CFD1, CHD1, CMP2, DPP1, EFT1, GCN20, GPH1, GPM1, GPP1, HAL5, IRE1, MEC1, MSS4, PFK2, PGK1, PPH21, PPT1, PRS3, PTC3, PTK2, REG2, RIM15, SAK1, SIW14, SNU114, TEL1, THI20, TUB2, VMA10, VMR1, YMR1, YPT52, YTA12, YTA7
RNA binding	32	CDC33, CRM1, DED1, GAR1, LSM2, NPL3, PCF11, PET54, PRP8, PRP9, RPL11A, RPL13A, RPL15A, RPL16A, RPL16B, RPL22A, RPL22B, RPL24A, RPL25, RPL36B, RPL37A, RPL6B, RPL9B, RPS14B, RPS4A, RPS4B, RPS9B, SBP1, SCP160, TIF1, UTP20, UTP21
ATP binding	29	ALA1, APC4, ATP3, BRR2, CCT6, CDC27, CFD1, CHD1, DED1, DUR1,2, GCN20, IDH1, LSC1, MCM6, PFK2, PGK1, PYC2, QNS1, RRM3, SNU114, TIF1, UBC4, UBR1, UFD4, VMA10, VMR1, YPT52, YTA12, YTA7
stress response	27	ALD6, CCT6, CIS3, CMP2, CPR5, CYC1, GPP1, HAL5, HOR7, HYR1, PAU10, PIR3, PRE3, RIM15, RPN10, SED1, SIR4, SIW14, SSZ1, TIP1, TIR3, TRM9, UBC4, UFD2, VHS3, YTA12, ZEO1
C-compound and carbohydrate metabolism	26	ADH1, ADH4, ALD6, ATF1, CYM1, FKS3, GAS5, GLC3, GPH1, GPM1, GPP1, GSC2, HXT11, IDH1, IRE1, LSC1, LYS21, NRG1, PDA1, PDC1, PFK2, PGK1, PPH21, PYC2, TPI1, YUR1
protein targeting, sorting and translocation	26	ARF2, BMH1, BMH2, CHC1, CMP2, CPR5, CRM1, CYM1, MFT1, MIA40, MLP2, NMD5, NPL3, NPL4, NUP116, PEP1, RDL1, RNA1, SNC1, SYN8, TIM54, UBX2, VMA3, VPS8, YPT52, YRB1
cell wall	22	CCW12, CCW22, CHS2, CIS3, CMP2, CWP2, DFG16, ECM30, FKS3, GAS5, GSC2, GSF2, HAL5, HPF1, PIR3, PPH21, RGT2, SED1, TIP1, VMA1, YUR1, ZEO1
nucleotide/nucleoside/nucleobase binding	22	ALA1, ARF2, BCD1, BRR2, CFD1, CHD1, DED1, EFT1, GCD11, GPH1, HNT1, MFT1, PGK1, QNS1, RFC1, SAR1, SNU114, TIF1, TUB2, VMR1, YPT52, YTA12
rRNA processing	22	BRR2, CHD1, DED1, DIS3, EBP2, ERB1, GAR1, LSM2, NPL3, PPH21, RNA1, RPL30, RPP1, RPS4A, RPS4B, RRP6, SBP1, TIF1, TSR1, UTP20, UTP21, YME2
DNA conformation modification (e.g. chromatin)	21	ARP9, ASF1, BDF2, CHD1, FKH1, HHF1, HTA2, HTB1, NHP10, NPT1, ORC3, PPH21, RIF1, RSC2, RXT3, SIN3, SIR4, SPT5, SUS1, SWI3, YNG1
development of asco- basidio- or zygospore	20	ADE16, BDF2, BMH1, BMH2, CDC33, DTR1, ERV14, FKS3, GSC2, IRA1, MDS3, MSS4, MUM2, PMD1, PRE3, PRT1, RIM21, SIN3, SSP1, UBC4
lipid, fatty acid and isoprenoid metabolism	19	ADH1, ALD6, ATF1, CAT2, ERG13, ERG2, ERG7, FAA4, FAS2, GPP1, NMA111, OSH3, PLB2, PLB3, PYC2, TEL1, TIP1, TPI1, VRG4
mitotic cell cycle and cell cycle control	19	CDC15, CDC33, CMP2, DIS3, DOM34, FIN1, FKH1, LTE1, MDM20, PPH21, PRP8, PRT1, RFC1, RPL10, SCP160, SDS24, TUB2, UBC4, YRB1
budding, cell polarity and filament formation	18	BMH1, BMH2, BNR1, BOI1, CHS2, CIS3, DFG10, DFG16, ERV14, FKH1, MSS4, NRG1, PAN1, PFY1, PIR3, PPH21, RIM21, SRO77
assembly of protein complexes	17	APC4, ARF2, CDC27, CFD1, FAS2, PAN1, RPL10, SAR1, SSP1, TUB2, UBC4, UBR1, UFD2, UFD4, USO1, VMA3, YTA12

**Table 3:** Categorisation of genes expressed exclusively in all four examined *S*. *cerevisiae* or S. *pastorianus* strains, respectively.

FunCat	*S*. *cerevisiae*		*S*. *pastorianus*		sum
	Count	Genes	Count	Genes	
Protein binding	49	APC1, APC2, CDC16, COG2, DBP1, DLD3, DYN2, END3, ENT2, HSP82, HST2, IOC2, LAT1, LOS1, MDJ1, NSG2, PMR1, PRE8, RPL11B, RPL23B, RPL33A, RPL40A, RPL40B, RPL42A, RPL43A, RPL5, RPS0B, RPS17B, RPS19B, RPS22B, RPS23A, RPS23B, RPS24A, RPS29A, RPS7B, SDH2, SEC8, SHY1, SMC2, SRP40, THI11, TIF6, UBC12, UBC6, UBC8, UBI4, UFD1, VPS1, ZDS2	31	AFG2, ARC40, ARP2, ARP3, CCT5, DBP5, DPM1, ENO1, HYP2, MCM4, MOT1, NOP58, NTF2, RAD50, RHO1, RPL12A, RPL13B, RPN5, RPS14A, RPS1A, RPS8A, RSP5, RTS1, SEC6, SKP1, SRP102, SSA2, SSE1, YAP1801, YPT6, ZDS1	80
ATP binding	18	APC1, APC2, BUD16, CDC16, CEM1, DBP1, DPS1, GRS2, HSP82, IRC5, PDR15, PMR1, RAD5, SMC2, STE6, UBC12, UBC6, UBC8	24	AFG2, ARP2, ARP3, AUS1, CCT5, DBP5, DDR48, DED81, ILS1, INO80, MCM4, MIS1, MOT1, PRP22, RAD50, RRP3, RSP5, SKP1, SSA2, SSE1, STH1, VMA2, YBT1, YPT6	42
Transcriptional control	19	ARP7, BUR6, BYE1, DBP1, FHL1, HST2, IRC5, KAR4, MED1, NUT1, OTU1, PHO4, PWP1, SIR1, SKG3, SPB1, SPP41, SPT8, SUT1	19	ARP2, ARP3, ELP2, HAP1, HHT1, INO80, MCM4, MOT1, REG1, RHO1, RRP3, RSC8, STB5, STH1, STP1, TAO3, TFG1, UPC2, YPT6	38
Phosphate metabolism	18	BUD16, FAB1, GLK1, GPP2, HIS2, HSP82, MKK2, NPP1, NPR1, PDR15, PHO8, PHO91, PIK1, RAD5, SAP155, SMC2, STE6, YEF1	19	AFG2, AUS1, DDR48, FRK1, HIS4, INO80, MCP2, PTC2, PTC5, RAD50, REG1, RHO1, RTS1, SSA2, SSK2, STH1, TDA1, YBT1, YPT6	37
Ribosome biogenesis	21	CGR1, EMG1, NOP15, RPL11B, RPL23B, RPL33A, RPL40A, RPL40B, RPL42A, RPL43A, RPL5, RPS0B, RPS19B, RPS22B, RPS23A, RPS23B, RPS24A, RPS29A, RPS7B, RSM19, TIF6	11	LOC1, LSG1, MRT4, RLP7, RPL12A, RPL13B, RPP1A, RPS14A, RPS1A, RPS8A, SNU13	32
C-compound and carbohydrate metabolism	17	AAD14, ALO1, CEM1, CIT2, CYB2, DLD3, GLK1, GPP2, *HXT3*, IDP2, ILV6, LAT1, LPD1, NAT3, PDC5, PDR15, SDH2	12	ACO1, ELO1, ENO1, GND1, IMA2, MNN9, NTH1, PGI1, PMT2, TKL1, TPS1, YAT1	29
Budding, cell polarity and filament formation	16	BSP1, BUD16, BUD27, BUD5, CRR1, END3, ENT2, HUA2, MKK2, NSG2, PIR1, RGA2, RPS0B, SAP155, SEC8, ZDS2	12	ARC40, ARP2, ARP3, ELO1, HSP150, MYO2, RHO1, SEC6, TAO3, YAP1801, YPT6, ZDS1	28
Ribosomal proteins	19	RPL11B, RPL23B, RPL33A, RPL40A, RPL40B, RPL42A, RPL43A, RPL5, RPS0B, RPS17B, RPS19B, RPS22B, RPS23A, RPS23B, RPS24A, RPS29A, RPS7B, RRP15, RSM19	8	MRPS8, RPL12A, RPL13B, RPP1A, RPS14A, RPS1A, RPS8A, SQT1	27
Stress response	15	FAB1, GPP2, HSP82, MDJ1, MKK2, PAU24, PIR1, PMR1, PRE8, RPL40A, RPL40B, TIR1, UBI4, YGP1, ZDS2	12	CCT5, DDR48, HSP150, NTH1, RPN5, RSP5, RTS1, SSA2, SSE1, SSK2, TPS1, ZDS1	27
Translation	18	DPS1, RPL11B, RPL23B, RPL33A, RPL40A, RPL40B, RPL43A, RPL5, RPS0B, RPS17B, RPS19B, RPS22B, RPS23A, RPS23B, RPS24A, RPS29A, RPS7B, TIF6	9	DBP5, DED81, HYP2, ILS1, RPL12A, RPL13B, RPS14A, RPS1A, RPS8A	27
Assembly of protein complexes	17	APC1, APC2, ATP22, BLM10, BSP1, CDC16, END3, ENT2, HUA2, LAT1, QCR2, RPS0B, SAC7, UBC12, UBC6, UBC8, UBI4	9	ARC40, ARP2, ARP3, RPN5, RSP5, SKP1, SQT1, SSA2, YAP1801	26
RNA binding	12	CBF5, DBP1, DIP2, EMG1, LOS1, MRD1, PUF3, RPL11B, RPL5, SRO9, TIF11, YRA1	13	DBP5, HRP1, IFM1, LOC1, NOP58, PRP22, RLP7, RPL12A, RPL13B, RPS14A, RRP3, UTP14, UTP5	25
rRNA processing	14	CBF5, CGR1, DBP1, DIP2, EMG1, FHL1, IFH1, MRD1, MTR3, POP1, RRP15, RRP17, SPB1, TIF6	10	DBP5, MRT4, NOP58, PRP22, RLP7, RRP3, SKI6, SNU13, UTP14, UTP5	24
Cell growth / morphogenesis	10	BSP1, CBF5, DBP1, ENT2, HUA2, KRE6, NSG2, RGA2, UBC8, ZDS2	10	ARC40, ARP2, ARP3, ELF1, ELO1, REG1, RHO1, TAO3, YAP1801, ZDS1	20
Mitotic cell cycle and cell cycle control	7	CBF5, CDC45, DIP2, MKK2, SMC2, SYF1, ZDS2	13	AFG2, ARP2, ARP3, MCM4, PTC5, RHO1, RSC8, SKP1, SPC97, SSA2, STH1, YPT6, ZDS1	20
DNA conformation modification (e.g. chromatin)	9	ARP7, HST2, IFH1, IOC2, IRC5, RSC58, SIR1, SPT8, ZDS2	10	ELF1, HHT1, INO80, NOP58, PNC1, RSC8, RSP5, STH1, YPT6, ZDS1	19
Protein targeting, sorting and translocation	7	ATG11, FAB1, MLP1, NIC96, NUP120, NUP188, VPS1	11	AFG2, ARP2, ARP3, NCE102, NTF2, SRP102, SSA2, TOM40, VPS13, VPS73, YPT6	18
Cell wall	8	ECM11, HLR1, KRE6, MKK2, PIR1, RLM1, SPE3, TIR1	9	ARP2, ARP3, HSP150, IFM1, MNN9, RHO1, SKI6, SSA2, SSK2	17
Proteasomal degradation (ubiquitin/proteasomal pathway)	13	APC1, APC2, BLM10, CDC16, DEF1, OTU1, PRE8, RPL40A, RPL40B, UBC12, UBC6, UBC8, UFD1	4	AFG2, RPN5, RSP5, SKP1	17
Nucleotide/nucleoside/nucleobase binding	5	BUD16, DBP1, IRC5, PMR1, VPS1	11	AFG2, DBP5, DED81, ILS1, MEF1, RHO1, RRP3, SSA2, SSE1, YBT1, YPT6	16
Lipid, fatty acid and isoprenoid metabolism	7	FAB1, GPP2, IDP2, ISC1, LPD1, NAT3, PIK1	8	ELO1, ERG4, ERG6, NCP1, OLE1, SLC1, UPC2, YAT1	15
Modification by ubiquitination, deubiquitination	11	APC1, APC2, CDC16, OTU1, RPL40A, RPL40B, UBC12, UBC6, UBC8, UBI4, UFD1	4	AFG2, CCT5, RSP5, SKP1	15
Pheromone response, mating-type determination, sex-specific proteins	9	END3, HSP82, KAR4, MDG1, RGA2, SIR1, STE6, UBC6, UBC8	6	AKR1, CSN12, ELO1, MYO2, RHO1, STH1	15
Protein transport	5	NIC96, NUP120, NUP188, PIR1, UFD1	10	AFG2, ARP2, ARP3, HSP150, NCE102, SRP102, SSA2, SXM1, TOM40, YPT6	15
Development of asco- basidio- or zygospore	9	GLK1, HSP82, PIK1, SPO14, SPO74, SPO77, UBC8, UBI4, YGP1	5	ARP2, ARP3, ELO1, LSG1, RSP5	14
Homeostasis of metal ions (Na, K, Ca etc.)	9	AHP1, GMC1, HIP1, IRC7, PDR15, PMR1, SPF1, SRO9, YEF1	4	AUS1, CTR3, SSA2, VCX1	13

Shown are the 20 MIPS FunCat categories with the highest gene count. Genes occurring only in one category are underlined. Highlighted in orange are the categories that are not among the top 20 categories of the other species.

**Table 4:** Categorisation of genes expressed in all four examined *S*. *cerevisiae* strains.

FunCat	*S*. *cerevisiae*	
	Count	Genes
Protein binding	49	APC1, APC2, CDC16, COG2, DBP1, DLD3, DYN2, END3, ENT2, HSP82, HST2, IOC2, LAT1, LOS1, MDJ1, NSG2, PMR1, PRE8, RPL11B, RPL23B, RPL33A, RPL40A, RPL40B, RPL42A, RPL43A, RPL5, RPS0B, RPS17B, RPS19B, RPS22B, RPS23A, RPS23B, RPS24A, RPS29A, RPS7B, SDH2, SEC8, SHY1, SMC2, SRP40, THI11, TIF6, UBC12, UBC6, UBC8, UBI4, UFD1, VPS1, ZDS2
Ribosome biogenesis	21	CGR1, EMG1, NOP15, RPL11B, RPL23B, RPL33A, RPL40A, RPL40B, RPL42A, RPL43A, RPL5, RPS0B, RPS19B, RPS22B, RPS23A, RPS23B, RPS24A, RPS29A, RPS7B, RSM19, TIF6
Ribosomal proteins	19	RPL11B, RPL23B, RPL33A, RPL40A, RPL40B, RPL42A, RPL43A, RPL5, RPS0B, RPS17B, RPS19B, RPS22B, RPS23A, RPS23B, RPS24A, RPS29A, RPS7B, RRP15, RSM19
Transcriptional control	19	ARP7, BUR6, BYE1, DBP1, FHL1, HST2, IRC5, KAR4, MED1, NUT1, OTU1, PHO4, PWP1, SIR1, SKG3, SPB1, SPP41, SPT8, SUT1
ATP binding	18	APC1, APC2, BUD16, CDC16, CEM1, DBP1, DPS1, GRS2, HSP82, IRC5, PDR15, PMR1, RAD5, SMC2, STE6, UBC12, UBC6, UBC8
Phosphate metabolism	18	BUD16, FAB1, GLK1, GPP2, HIS2, HSP82, MKK2, NPP1, NPR1, PDR15, PHO8, PHO91, PIK1, RAD5, SAP155, SMC2, STE6, YEF1
Translation	18	DPS1, RPL11B, RPL23B, RPL33A, RPL40A, RPL40B, RPL43A, RPL5, RPS0B, RPS17B, RPS19B, RPS22B, RPS23A, RPS23B, RPS24A, RPS29A, RPS7B, TIF6
Assembly of protein complexes	17	APC1, APC2, ATP22, BLM10, BSP1, CDC16, END3, ENT2, HUA2, LAT1, QCR2, RPS0B, SAC7, UBC12, UBC6, UBC8, UBI4
C-compound and carbohydrate metabolism	17	AAD14, ALO1, CEM1, CIT2, CYB2, DLD3, GLK1, GPP2, *HXT3*, IDP2, ILV6, LAT1, LPD1, NAT3, PDC5, PDR15, SDH2
Budding, cell polarity and filament formation	16	BSP1, BUD16, BUD27, BUD5, CRR1, END3, ENT2, HUA2, MKK2, NSG2, PIR1, RGA2, RPS0B, SAP155, SEC8, ZDS2
Stress response	15	FAB1, GPP2, HSP82, MDJ1, MKK2, PAU24, PIR1, PMR1, PRE8, RPL40A, RPL40B, TIR1, UBI4, YGP1, ZDS2
rRNA processing	14	CBF5, CGR1, DBP1, DIP2, EMG1, FHL1, IFH1, MRD1, MTR3, POP1, RRP15, RRP17, SPB1, TIF6
Proteasomal degradation (ubiquitin/proteasomal pathway)	13	APC1, APC2, BLM10, CDC16, DEF1, OTU1, PRE8, RPL40A, RPL40B, UBC12, UBC6, UBC8, UFD1
RNA binding	12	CBF5, DBP1, DIP2, EMG1, LOS1, MRD1, PUF3, RPL11B, RPL5, SRO9, TIF11, YRA1
Modification by ubiquitination, deubiquitination	11	APC1, APC2, CDC16, OTU1, RPL40A, RPL40B, UBC12, UBC6, UBC8, UBI4, UFD1
Cell growth / morphogenesis	10	BSP1, CBF5, DBP1, ENT2, HUA2, KRE6, NSG2, RGA2, UBC8, ZDS2
Development of asco- basidio- or zygospore	9	GLK1, HSP82, PIK1, SPO14, SPO74, SPO77, UBC8, UBI4, YGP1
DNA conformation modification (e.g. chromatin)	9	ARP7, HST2, IFH1, IOC2, IRC5, RSC58, SIR1, SPT8, ZDS2
Homeostasis of metal ions (Na, K, Ca etc.)	9	AHP1, GMC1, HIP1, IRC7, PDR15, PMR1, SPF1, SRO9, YEF1
Pheromone response, mating-type determination, sex-specific proteins	9	END3, HSP82, KAR4, MDG1, RGA2, SIR1, STE6, UBC6, UBC8

Shown are the 20 MIPS FunCat categories with the highest gene count. Genes occurring only in one category are underlined.

**Table 5:** Categorisation of genes expressed in all four examined *S*. *pastorianus* strains.

FunCat	*S*. *pastorianus*	
	Count	Genes
Protein binding	31	AFG2, ARC40, ARP2, ARP3, CCT5, DBP5, DPM1, ENO1, HYP2, MCM4, MOT1, NOP58, NTF2, RAD50, RHO1, RPL12A, RPL13B, RPN5, RPS14A, RPS1A, RPS8A, RSP5, RTS1, SEC6, SKP1, SRP102, SSA2, SSE1, YAP1801, YPT6, ZDS1
ATP binding	24	AFG2, ARP2, ARP3, AUS1, CCT5, DBP5, DDR48, DED81, ILS1, INO80, MCM4, MIS1, MOT1, PRP22, RAD50, RRP3, RSP5, SKP1, SSA2, SSE1, STH1, VMA2, YBT1, YPT6
Phosphate metabolism	19	AFG2, AUS1, DDR48, FRK1, HIS4, INO80, MCP2, PTC2, PTC5, RAD50, REG1, RHO1, RTS1, SSA2, SSK2, STH1, TDA1, YBT1, YPT6
Transcriptional control	19	ARP2, ARP3, ELP2, HAP1, HHT1, INO80, MCM4, MOT1, REG1, RHO1, RRP3, RSC8, STB5, STH1, STP1, TAO3, TFG1, UPC2, YPT6
Mitotic cell cycle and cell cycle control	13	AFG2, ARP2, ARP3, MCM4, PTC5, RHO1, RSC8, SKP1, SPC97, SSA2, STH1, YPT6, ZDS1
RNA binding	13	DBP5, HRP1, IFM1, LOC1, NOP58, PRP22, RLP7, RPL12A, RPL13B, RPS14A, RRP3, UTP14, UTP5
Budding, cell polarity and filament formation	12	ARC40, ARP2, ARP3, ELO1, HSP150, MYO2, RHO1, SEC6, TAO3, YAP1801, YPT6, ZDS1
C-compound and carbohydrate metabolism	12	ACO1, ELO1, ENO1, GND1, IMA2, MNN9, NTH1, PGI1, PMT2, TKL1, TPS1, YAT1
Stress response	12	CCT5, DDR48, HSP150, NTH1, RPN5, RSP5, RTS1, SSA2, SSE1, SSK2, TPS1, ZDS1
Nucleotide/nucleoside/nucleobase binding	11	AFG2, DBP5, DED81, ILS1, MEF1, RHO1, RRP3, SSA2, SSE1, YBT1, YPT6
Protein targeting, sorting and translocation	11	AFG2, ARP2, ARP3, NCE102, NTF2, SRP102, SSA2, TOM40, VPS13, VPS73, YPT6
Ribosome biogenesis	11	LOC1, LSG1, MRT4, RLP7, RPL12A, RPL13B, RPP1A, RPS14A, RPS1A, RPS8A, SNU13
Cell growth / morphogenesis	10	ARC40, ARP2, ARP3, ELF1, ELO1, REG1, RHO1, TAO3, YAP1801, ZDS1
DNA conformation modification (e.g. chromatin)	10	ELF1, HHT1, INO80, NOP58, PNC1, RSC8, RSP5, STH1, YPT6, ZDS1
Protein transport	10	AFG2, ARP2, ARP3, HSP150, NCE102, SRP102, SSA2, SXM1, TOM40, YPT6
rRNA processing	10	DBP5, MRT4, NOP58, PRP22, RLP7, RRP3, SKI6, SNU13, UTP14, UTP5
Assembly of protein complexes	9	ARC40, ARP2, ARP3, RPN5, RSP5, SKP1, SQT1, SSA2, YAP1801
Cell wall	9	ARP2, ARP3, HSP150, IFM1, MNN9, RHO1, SKI6, SSA2, SSK2
Translation	9	DBP5, DED81, HYP2, ILS1, RPL12A, RPL13B, RPS14A, RPS1A, RPS8A
Lipid, fatty acid and isoprenoid metabolism	8	ELO1, ERG4, ERG6, NCP1, OLE1, SLC1, UPC2, YAT1

Shown are the 20 MIPS FunCat categories with the highest gene count. Genes occurring only in one category are underlined.

## Conclusions

The data set and bioinformatics workflow obtained in this study demonstrates the potential of comparative *de novo* transcriptomics to differentiate *Saccharomyces* brewing yeasts into categories beyond the general top and bottom fermenting *S*. *cerevisiae* and *S*. *pastorianus* yeasts, even without their established genome sequences. The transcriptomic profiles obtained in our experimental setting do not necessarily reflect brewers experiences in their performance and metabolic behaviour. In the transcriptomic analysis Ale yeasts presented themselves as less diverse compared to lager yeasts, which supports the reported low genetic variation between *S*. *cerevisiae* isolates [12]. While two distinct phenotypic groups have been described for *S pastorianus* [8], we could identify three distinct clusters with the Frohberg yeasts clustering together. Actually, this provides the intended independent view on the classification of these yeasts. It identifies different groups of transcripts in groups of different brewing types of yeasts, which may enable their recognition and a possible (re)definition of brewing types beyond brewers’ experience. While this study is a proof of concept so far for the demonstration of the potential of the *de novo* transcriptomic approach, it enables the in-depth exploration of the core and accessory gene expression profiles and concomitant metabolic differences of *Saccharomyces* brewing yeasts.

## Supporting information

S1 File Helmholz MIPS database. (ZIP)
